# Testing the importance of jasmonate signalling in induction of plant defences upon cabbage aphid (*Brevicoryne brassicae*) attack

**DOI:** 10.1186/1471-2164-12-423

**Published:** 2011-08-19

**Authors:** Anna Kuśnierczyk, Diem HT Tran, Per Winge, Tommy S Jørstad, John C Reese, Joanna Troczyńska, Atle M Bones

**Affiliations:** 1 Department of Biology, The Norwegian University of Science and Technology, Realfagbygget, 7491 Trondheim, Norway; 2 Scanpower AS, 7462 Trondheim, Norway; 3 Department of Entomology, Kansas State University, Manhattan, KS 66506, USA; 4 Department of Plant Physiology, University of Technology and Agriculture, Bernardyńska 6, 85-029 Bydgoszcz, Poland

**Keywords:** aphid, gene expression, infestation, jasmonic acid signalling, microarrays, plant defence, EPG

## Abstract

**Background:**

Phloem-feeding aphids deprive plants of assimilates, but mostly manage to avoid causing the mechanical tissue damage inflicted by chewing insects. Nevertheless, jasmonate signalling that is induced by infestation is important in mediating resistance to phloem feeders. Aphid attack induces the jasmonic acid signalling pathway, but very little is known about the specific impact jasmonates have on the expression of genes that respond to aphid attack.

**Results:**

We have evaluated the function that jasmonates have in regulating *Arabidopsis thaliana *responses to cabbage aphid (*Brevicoryne brassicae*) by conducting a large-scale transcriptional analysis of two mutants: *aos*, which is defective in jasmonate production, and *fou2*, which constitutively induces jasmonic acid biosynthesis. This analysis enabled us to determine which genes' expression patterns depend on the jasmonic acid signalling pathway. We identified more than 200 genes whose expression in non-challenged plants depended on jasmonate levels and more than 800 genes that responded differently to infestation in *aos *and *fou2 *plants than in wt. Several aphid-induced changes were compromised in the *aos *mutant, particularly genes connected to regulation of transcription, defence responses and redox changes. Due to jasmonate-triggered pre-activation of *fou2*, its transcriptional profile in non-challenged plants mimicked the induction of defence responses in wt. Additional activation of *fou2 *upon aphid attack was therefore limited. Insect fitness experiments revealed that the physiological consequences of *fou2 *mutation contributed to more effective protection against *B. brassicae*. However, the observed resistance of the *fou2 *mutant was based on antibiotic rather than feeding deterrent properties of the mutant as indicated by an analysis of aphid feeding behaviour.

**Conclusions:**

Analysis of transcriptional profiles of wt, *aos *and *fou2 *plants revealed that the expression of more than 200 genes is dependent on jasmonate status, regardless of external stimuli. Moreover, the aphid-induced response of more than 800 transcripts is regulated by jasmonate signalling. Thus, in plants lacking jasmonates many of the defence-related responses induced by infestation in wt plants are impaired. Constant up-regulation of jasmonate signalling as evident in the *fou2 *mutant causes reduction in aphid population growth, likely as a result of antibiotic properties of *fou2 *plants. However, *aos *mutation does not seem to affect aphid performance when the density of *B. brassicae *populations on plants is low and aphids are free to move around.

## Background

Jasmonates, including jasmonic acid (JA) and the biologically active intermediates and derivatives of the JA biosynthetic pathway, are powerful regulators of plant development and inducible resistance. By mediating signal transduction they influence changes in expression profiles of a wide range of genes involved in plant defence [1]. Induction of JA-related response has often been linked to tissue damage, and the important roles of JA signalling in defence against bacterial and fungal infections or caterpillar attack are well documented (for reviews [2-4]). More recent research, however, provides evidence for the activation of JA-mediated defence upon attack by phloem-sucking insects, such as aphids and silverleaf whitefly nymphs, which try to avoid tissue damage during feeding [5-10]. Phloem feeders possess stylet-like mouthparts, which they use to ingest phloem sap. During penetration of plant tissue the stylet is manoeuvred through plant tissue until it is finally anchored in a sieve tube element. Here it can stay for several hours or even days, facilitating a continuous sap supply. By avoiding extensive tissue wounding, aphids minimize the risk of inducing defence responses in the attacked plant while depriving it of assimilates. In the case of a massive infestation, the loss of nutrients interferes with plant growth and development, and may eventually lead to plant death. Constitutive or transient activation of JA-related responses is known to enhance a plant's resistance to phloem feeders, including aphids [11-13].

JA is biosynthesized from polyunsaturated fatty acids released from chloroplast membranes via a series of enzymatic reactions usually referred to as the octadecanoid pathway. In pathogen-free laboratory conditions, a non-functional JA pathway does not result in any disturbance in normal vegetative growth. In a more natural environment, however, mutant plants that do not synthesize JA are more susceptible to pathogen attack because they fail to activate JA-dependent defences [14]. A knock-out mutation of the *allene oxide synthase *(*AOS*) gene, whose product is an enzyme essential for the synthesis of 12-oxophytodienoic acid (OPDA), a precursor for the synthesis of JA, results in a phenotype unable to produce JA or any JA derivatives [15] (Additional file 1 Figure S1). *AtAOS *is a single-copy gene, and no alternative enzymes possessing the same catalytic activity have been found in *Arabidopsis *[16]. Thus, the induction of JA-dependent genes is impaired in the *aos *mutant [15].

The *fatty acid oxygenation up-regulated 2 *(*fou2*) mutant was isolated by Bonaventure and co-workers in a search for plants with increased activity of two key JA biosynthetic enzymes: lipoxygenase (LOX) and AOS. JA and OPDA levels are almost doubled in non-challenged *fou2 *plants compared to wt [17] (Additional file 1 Figure S1). The *fou2 *allele carries a missense mutation resulting in an amino acid substitution in the Two Pore Channel 1 (TPC1) protein (encoded by *At4g03560*) [17]. TPC1 forms a non-specific, slowly activating, Ca^2+^-regulated cation channel in vacuolar membranes [18]. In *fou2 *the TPC1 channel has different electrophysiological properties: lower voltage is required for its activation and its time-dependent conductivity is higher than in wt [17]. Probably due to the increased sensitivity of voltage sensors in the mutated TPC1, the activation of the JA biosynthetic pathway upon wounding is stronger in *fou2 *plants and the levels of free JA and OPDA are higher in the mutant relative to wt [17].

Transcriptional analyses of aphid-infested *Arabidopsis *plants have revealed substantial changes in the expression profiles of many defence-related genes [7,9,19-21]. Several genes whose products are involved in JA synthesis or JA-dependent signalling have been reported to be up-regulated, indicating that JA-derived compounds play a role in the regulation of expressional changes. As a result of transcriptional reprogramming, the production of proteins involved in defence is promoted [22] and the metabolite profiles of plants are changed [7,23-25]. Despite significant progress in our understanding of plant responses triggered by phloem feeders attack (for reviews: [26-30]), it is largely unknown how much the induction of these defences relies on JA signalling.

In this study, we provide new insights into the role of jasmonates in the regulation of defence responses upon aphid attack. A specialized phloem feeder is represented by the cabbage aphid, *Brevicoryne brassicae*, for which a model of *Arabidopsis*-aphid interactions has been well established [8]. Our aim is to identify the genes whose expressional changes are controlled by JA signalling. The subsequent parts of this work concentrate on the following problems: Which genes are primarily dependent on jasmonates for their expression? How is the aphid-induced plant defence affected by the absence of JA or the constitutive up-regulation of the JA pathway? How does the impact of the *aos *and *fou2 *mutations affect aphid performance? To address these problems we have performed transcriptional profiling of both aphid-challenged and non-challenged wild type plants as well as *aos *and *fou2 *mutants using full genome oligonucleotide microarrays. Further, insect fitness experiments and Electrical Penetration Graph analysis have been undertaken to determine how the JA status of the host plants influences the survival and behaviour of insects.

## Results

**Figure 1:**
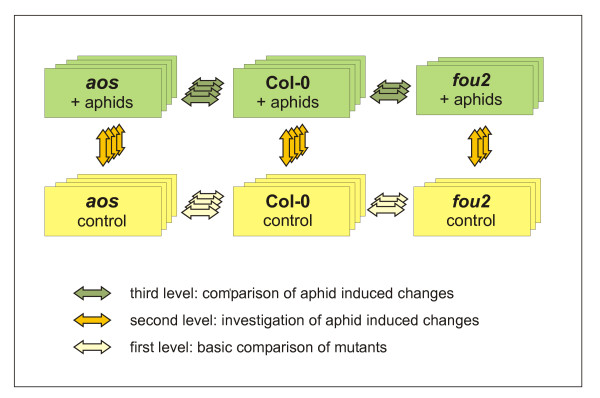
**Microarray experimental design**. Each square represents one biological replicate consisting of control, non-infested plants (yellow) or plants that have been subjected to 72 hours of infestation with *Brevicoryne brassicae *(green). Each arrow represents a direct comparison with the use of one microarray slide. The experiment was designed to assess three levels of comparisons: the first explores basic transcriptional profiles of non-infested mutants vs. wt plants (yellow arrows), the second aims to measure gene expression changes after aphid attack (orange arrows), while the third investigates the differences in the transcriptional reprogramming of mutants and wt plants (green arrows).

To investigate the importance of JA signalling in transcriptional reprogramming of *A. thaliana *triggered by aphid attack, we designed an experiment that included comparisons of genome-wide transcription profiles at three levels (Figure 1). Each level was comprised of a series of microarray hybridizations exploring transcriptional changes in at least three biological replicates per comparison. At the first level, which we regard as the basic comparison, we aimed to identify and classify genes that are dependent on jasmonates for their basic expression. This was done by comparing the transcription profiles of non-challenged wt plants and the two mutants, *aos *and *fou2*. At the second level the changes in transcriptional activity resulting from 72 h of aphid infestation of wt, *aos *and *fou2 *plants were analysed in each of the three lines independently. At the third level we directly compared aphid-induced transcriptional changes in each of the mutants with the corresponding changes in wt plants. The microarray data generated at all three levels were used in the statistical analysis. Twelve genes that were particularly interesting due to their involvement in JA signalling and/or their association with plant defence responses were further selected for qRT-PCR analysis. The gene expression profiles revealed by qRT-PCR analysis seem to correspond well to the profiles obtained from microarray data (Additional file 2 Figure S2).

### Identification of genes regulated by the JA signalling pathway

**Figure 2:**
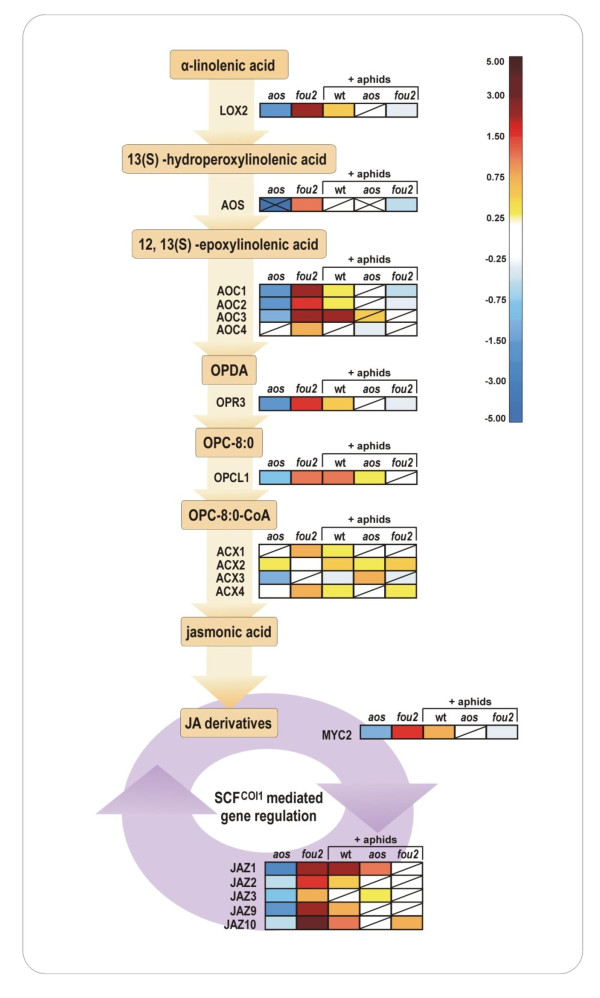
**Regulation of genes involved in the JA biosythesis pathway**. The colour code in squares indicates gene expression changes in *aos *and *fou2 *mutants in comparison to wt and changes in *B. brassicae *infested wt, *aos *and *fou2 *versus aphid free control plants of the corresponding genotype. A diagonal line inside a square indicates that gene regulation was not statistically significant. Two crossing diagonal lines indicate that the *AOS *gene is knockout in the *aos *mutant. The colour scale represents log_2 _transformed gene expression ratios. Abbreviations: *aos*, gene expression profiles of *aos *mutant in comparison to wt; *fou2*, gene expression profiles of *fou2 *mutant in comparison to wt; [wt, *aos*, *fou2*] + aphids, aphid-mediated changes in gene expression profiles in wt, *aos *and *fou2*, respectively. LOX2, LIPOXYGENASE 2; AOS, ALLENE OXIDE SYNTHASE; AOC, ALLENE OXIDE CYCLASE; OPDA, (9S,13S)-12-oxo-*cis*-10,15-phytodienoic acid; OPR3, OPDA REDUCTASE3; OPC-8:0, 3-oxo-2-(cis-2'-pentenyl)-cyclopentane-1-octanoic acid (OPC-8:0); OPCL1, OPC-8:0 CoA LIGASE; ACX, OPC-8:0 CoA OXIDASE; MYC2, JASMONATE INSENSITIVE1 transcription factor; JAZ, JASMONATE-ZIM-DOMAIN PROTEIN; SCF^COI1^, SCF ubiquitin E3 ligase-CORONATINE INSENSITIVE1 (COI1) complex.

Both *aos *and *fou2 *mutations have a great impact on the regulation of the JA biosythesis pathway regardless of environmental conditions (Figure 2). Therefore, before investigation of genes whose transcriptional regulation in response to *B. brassicae *attack is controlled by JA signalling, we aimed to identify the genes whose basic expression in non-challenged plants is modified according to endogenous JA levels. The following criteria have been adopted to identify jasmonate-dependent genes. To be considered positively regulated by jasmonates, a gene had to be down-regulated in *aos *(log2 ratio < -0.5) and up-regulated in *fou2 *(log2 ratio > 0.5) as compared to wt. Conversely, the expression of genes classified as negatively regulated by jasmonates was positively affected in *aos *and negatively affected in *fou2*, respectively. One-hundred seventy-two genes were found to be positively regulated by jasmonates and have been classified into the following functional gene classes: transcripts involved in JA synthesis and JA signalling, defence-related proteins including myrosinases and myrosinase binding or associated proteins, genes whose products are involved in the regulation of transcription, redox balance, cell wall modification, protein modification, nucleoside/nucleotide metabolism, transport and lipid metabolism (Additional file 3 Table S1). Among the 39 genes whose expression was negatively regulated by jasmonates were several transcription regulators, genes coding for proteins with ankyrin repeats and connected to redox status. Except for genes with unknown functions, other categories were represented by only 1-2 members (Additional file 4 Table S2).

As JA signalling is important in the regulation of plant defensive responses triggered by aphid attack we expected to observe the effect of the changed JA status on the expression of aphid-responsive genes. It should be noted that not all genes classified by us as JA dependent were found to be responsive to *B. brassicae *attack. Although a number of JA-dependent genes were induced by *B. brassicae *in wt plants, their aphid-mediated induction was impaired not only in *aos*, as expected, but also in *fou2 *plants. This was the case for several transcripts whose products are involved either in the biosynthesis of JA or in JA-mediated signalling (Figure 2), defence-related genes, transcription factors and redox homeostasis. Table 1 summarizes expression profiles of all genes that have been classified by us as JA dependent and whose responsiveness to *B. brassicae *attack was changed in *aos *or *fou2 *mutants relative to wt.

**Table 1:** Jasmonate-dependent genes whose responsiveness to *B. brassicae *attack was changed in *aos *or *fou2 *mutants relative to wt.

				Infested with *Brevicoryne brassicae*		
				
Gene	Accession	*aos*/wt	*fou2*/wt	wt*B*/wt	*aosB*/*aos*	*fou2B*/*fou2*
**JA synthesis**						
LOX2	At3g45140	-1.97	2.09	0.55	NS	-0.42
AOC3	At3g25780	-1.26	2.36	2.19	NS	NS
OPR3	At2g06050	-1.51	1.62	0.52	NS	-0.47
OPCL1	At1g20510	-0.77	1.13	1.08	0.29	NS
**JA signalling**						
CORI3	At4g23600	-1.60	2.42	0.83	NS	NS
MYC2 (JIN1)	At1g32640	-1.45	1.86	0.91	NS	-0.43
JAZ1	At1g19180	-2.31	2.62	2.10	1.15	NS
JAZ2	At1g74950	-0.55	1.89	0.69	NS	NS
JAZ6	At1g72450	-1.41	2.00	0.58	NS	NS
JAZ9	At1g70700	-1.99	2.65	0.79	NS	NS
JAZ10	At5g13220	-0.85	3.69	1.10	NA	0.91
**Defence**						
PDF1.2	At5g44420	-3.33	3.53	2.99	NS	NS
PDF1.2b	At2g26020	-3.53	3.31	3.00	NS	NS
PDF1.3	At2g26010	-3.46	3.23	2.80	NS	NS
PDF1.2c	At5g44430	-3.32	3.23	2.70	NS	NS
AFP1	At1g75830	-3.04	3.40	2.64	NS	NS
S-adenosylmethionine-dependent methyltransferase	At3g44870	-1.45	3.13	1.91	NS	NS
MBP1	At1g52040	-2.53	4.98	0.73	NS	NS
S-adenosylmethionine-dependent methyltransferase	At3g44860	-1.27	2.60	0.98	NS	-0.86
arginase	At4g08870	-1.44	4.05	0.94	NS	-0.63
strictosidine synthase	At3g51450	-1.18	1.70	0.55	-1.01	NS
EDS5	At4g39030	-0.52	0.87	1.87	1.15	NS
ASA1	At5g05730	-0.70	0.73	1.08	0.64	0.53
TAT3	At2g24850	-1.58	3.52	4.20	2.03	NS
CYP79B2	At4g3995	-0.83	1.17	1.41	1.15	0.47
PR4	At3g04720	-0.81	1.35	2.32	0.76	1.13
trypsin inhibitor 1 (ATTI1)	At2g43510	-1.08	3.71	1.74	0.86	1.14
trypsin inhibitor	At1g73260	-1.65	2.85	1.00	1.44	1.62
protease inhibitor (LTP)	At5g48490	-0.64	0.79	-0.79	-0.67	-0.98
HSP17.4-CIII	At1g54050	-0.95	0.81	-0.67	-0.59	-0.55
**Transcription factors**						
WRKY75	At5g13080	-1.82	2.52	3.23	3.18	1.31
ERF2	At5g47220	-1.13	0.88	2.06	1.02	0.42
RHL41/ZAT12	At5g59820	-1.39	1.85	3.02	2.26	NS
HSF6	At5g62020	-0.90	1.05	0.64	0.81	NS
**Redox**						
Atperox P37	At4g08770	-1.46	1.08	1.57	1.36	1.41
GST22/ATGSTU4	At2g29460	-0.73	0.99	1.87	1.20	1.05
MDAR4	At5g03630	-0.75	0.62	0.67	0.44	NS
Atperox P32	At3g32980	-1.49	1.38	-0.82	0.43	NS
FRO6	At5g49730	-0.84	0.70	-0.76	0.46	-1.25
copper amine oxidase	At1g31710	-1.56	1.00	-1.17	0.65	NS
**Auxin synthesis**						
ILL4	At1g51760	-0.70	1.71	1.84	NS	NS
NIT2	At3g44300	-0.82	2.10	1.20	0.95	1.68
**cell wall modification**						
PGIP2	At5g06870	-0.90	2.72	0.63	NS	0.70
AGP	At1g03820	-0.55	2.81	NS	0.57	1.53
FLR1	At3g12145	-0.91	2.04	-0.58	0.53	0.60
invertase/pectin methylesterase inhibitor	At1g62770	-1.30	0.87	-1.01	1.63	1.18
**lipid metabolism**						
esterase/lipase/thioesterase family protein	At2g39420	-0.77	2.29	1.60	NS	NS
**unknown**						
unknown plant specific protein (AR781)	At2g26530	-0.53	1.36	1.31	0.39	NS

The values in the table represent log_2 _transformed gene expression changes for the following comparisons: *aos*/wt, change in a gene expression level in *aos *mutant in comparison to wt; *fou2*/wt, change in a gene expression level in *fou2 *mutant in comparison to wt; wtB/wt, change in a gene expression level in wt plants attacked by aphids in comparison to aphid-free wt controls; *aos*B/*aos*, change in a gene expression level in *aos *plants attacked by aphids in comparison to aphid-free *aos *controls; *fou2*B/*fou2*, change in a gene expression level in *fou2 *plants attacked by aphids in comparison to aphid-free *fou2 *controls; NS, not statistically significantly regulated.

### JA signalling has an overall significant impact on the regulation of *Arabidopsis thaliana *responses to *Brevicoryne brassicae *attack

**Figure 3:**
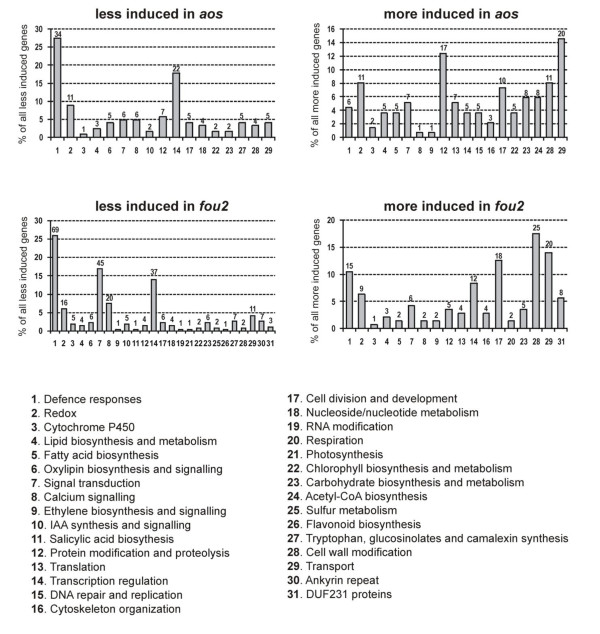
**General overview of differences in responsiveness of *aos *and *fou2 *mutants to *B. brassicae *attack compared to responsiveness in wt**. Bars represent contribution of different functional categories in the pool of all genes that were either less or more induced upon infestation with aphids in *aos *or *fou2 *genotype in comparison to wt. Numbers placed on the top of each bar indicate how many genes were differentially regulated in response to *B. brassicae *attack in each functional category in a given mutant as compared to wt.

**Figure 4:**
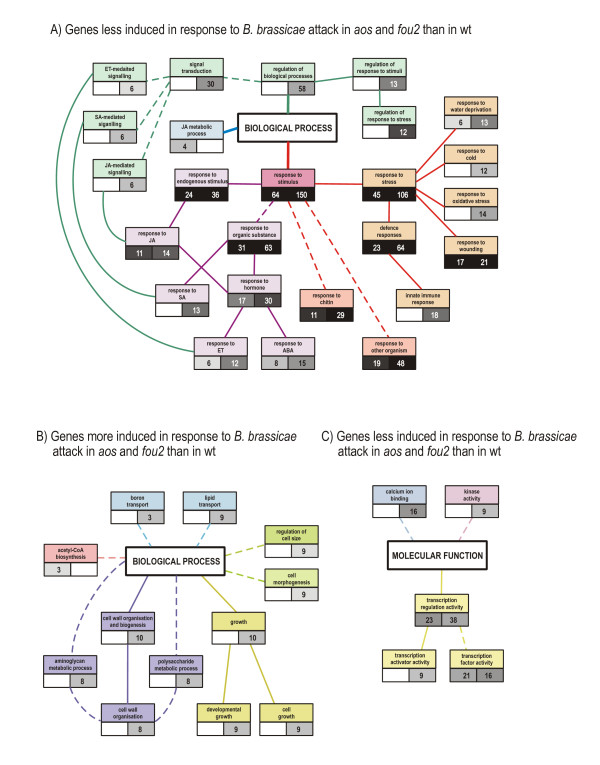
**Simplified graphic representation of enriched GO terms connected to biological process or molecular function in genes that were less (A, C) or more (B) induced compared to wt in their response to *Brevicoryne brassicae *attack in *aos *and *fou2 *mutants**. The graph is based on results generated by AmiGO Term Enrichment [31] of functional gene networks. Functionally connected GO categories are represented with the same colour code. Streaked lines indicate that GO terms that exist between the two connected GO terms were omitted from presentation for clarity reasons. Only GO terms classified as enriched according to AmiGO Term Enrichment (with *p *value < 0.05) are presented in the graph. The numbers of genes attributed to a given GO term for *aos *and *fou2 *mutants are indicated in the left and right boxes under given GO terms, respectively. A darker shade of grey corresponds to higher significance of enrichment (lower *p *value) of a given GO term according to the AmiGO Term Enrichment analysis.

Among all aphid responsive genes that have been classified as JA dependent in non-infested plants, the majority were found to have altered responsiveness to *B. brassicae *attack in the mutants compared to wt (Table 1). However, several other genes that did not change expression in non-challenged *aos *and *fou2 *displayed unique responses to aphid infestation in the mutant plants. A list of genes responding differently to *B. brassicae *attack in a given mutant was created based on the following criteria: (i) the aphid-induced regulation of a given gene had to be statistically significant for at least one of the two compared genotypes (e.g. for the given mutant or for wt); (ii) the difference in the aphid-induced gene regulation (expressed in log_2 _ratio) between the two compared genotypes had to be larger than one. The complete lists of genes fulfilling these requirements are presented in Additional files 5, 6, 7, 8 Tables S3, S4, S5 and S6 while Figure 3 represents the distribution of functional categories among the differentially responding genes in the two mutants. Although, as expected, the aphid-induced responsiveness of many genes was changed in the mutants relative to wt, the direction of the observed changes was surprisingly similar in the *aos *and *fou2 *mutants. For example, the relatively large groups of genes related to defence and regulation of transcription were less responsive to infestation both in *aos *and *fou2 *(Figure 3). Similarly, among genes identified as more responsive to aphids in the mutants than in wt, transcripts connected to transport, cell wall modification, cell division and development and cytoskeleton organisation were more induced in both mutants (Figure 3). To evaluate an overall impact of the *aos *and *fou2 *mutations on the different functional gene categories of aphid-responsive genes, GO Term Enrichment analysis was performed with the use of AmiGO Term Enrichment software [31]. Four sets of genes that responded differentially to *B. brassicae *infestation (corresponding to Additional files 5, 6, 7, 8 Tables S3, S4, S5 and S6) were annotated with Gene Ontology terms and AmiGo was used to determine whether the observed levels of annotation for the particular sets were significant in the context of a background set (i.e. all *A. thaliana *genes that have been attributed to a particular GO term). The statistically significantly overrepresented GO terms connected to Biological Process and Molecular Function nodes were then visualized according to significance level and the numbers of genes attributed to linked GO terms were given separately for *aos *and *fou2 *mutants (Figure 4).

### *B. brassicae *induced regulation of transcription factors and defence-related genes is largely controlled by JA signalling

The JA signalling pathway is believed to significantly contribute to the regulation of defence-connected genes under stress conditions. The GO terms denoted "transcription regulation activity" and "response to stress" with the sub-nodes "defence responses" and "response to wounding" were statistically significantly overrepresented among genes less responsive to aphid attack both in *aos *and *fou2 *mutants (Figure 4A, C). These categories taken together contributed almost half of the genes whose responsiveness was negatively affected in *aos *and *fou2 *plants (Figure 3). Although the majority of the genes that responded to *B. brassicae *infestation in wt plants were induced in the challenged *aos *as well, their regulation was weaker in the mutant than in wt (Additional file 5 Table S3). Twenty two genes, whose products are involved in regulation of transcription and 34 transcripts connected to defence showed no induction or weaker up-regulation upon infestation in the *aos *mutant. Several transcription factors and defence-related proteins were, in contrast to wt, either not induced or down-regulated in the aphid-challenged *aos *plants; i.e. *BTB and TAZ domain protein 5 *(*BT5*), *dehydration-responsive element-binding protein 2A *(*DREB2A*), *ethylene-responsive transcription factors ERF11 *and *ERF13*, *myb family transcription factor *(*MYB50*), *C2H2 type family protein*, *DARK INDUCIBLE 11 *(*DIN11*), *sulfotransferase family protein *(*At5g07010*), *strictosidine synthase*, *plant defensine 1 *(*PDF1*), *cysteine-rich antifungal protein 1 precursor *(*AFP1*), *heat shock protein 81-1 *(*HSP81-1*) and *arginase*. These observations clearly show that JA signalling is important in the activation of defensive responses triggered by *B. brassicae *attack. However, the fact that some genes were up-regulated during infestation despite of the lack of AOS enzyme activity indicates that JA signalling is, as expected, not the only system controlling gene regulation. Interestingly, some of the defence-related transcripts (e.g. *PR1*, *HR3*, *disease resistance genes*: *At1g57630*, *At3g25010*, *At2g47800*) accumulated in the non-challenged *aos *plants as compared to wt, probably as a result of stress connected to the lack of JA or an imbalance between JA and SA signalling pathways.

In the *fou2 *mutant, several transcription factors and defence-related genes were already up-regulated in non-challenged plants compared to wt, indicating constant activation of defence caused by the increased endogenous JA levels [e.g. *WRKY, ethylene responsive transcription factors, zinc finger family proteins, pathogenesis related proteins PR1 and PR2, enhanced disease susceptibility 5 *(*EDS5*)*, protease inhibitors, cysteine-rich antifungal proteins: PDF1.1, PDF1.2, PDF1.2b PDF1.2c, PDF1.3, DARK INDUCIBLE 11 *(*DIN11*)]. Often the induction of these genes was stronger in non-challenged *fou2 *mutants in comparison to wt than in the infested wt compared to aphid free wt. In such cases no additional induction was noted in the aphid-attacked *fou2 *mutant compared to the aphid-free *fou2 *control. For other genes a slight additional induction of already up-regulated transcripts was observed in *fou2 *plants attacked by *B. brassicae *(Additional file 7 Table S5). Out of 41 transcription factors and 74 defence-related genes up-regulated upon *B. brassicae *infestation in wt, but having changed aphid-triggered regulation in one or both mutants, 37 and 69 genes, respectively, were less up-regulated or not induced in the *fou2 *mutant in response to infestation. These results indicate that the activation of defence responses may have an overall induction threshold. A potential for an additional, aphid-triggered induction is likely limited when the basal activation of transcripts in non-challenged *fou2 *plants is already very high.

Several senescence-associated genes responded to aphid attack with strong induction. Overall, the intensity of aphid-induced changes in this group of genes was similar in wt and *aos *plants, but slightly weaker in the *fou2 *mutant. Thus JA signalling seems not to be the key factor controlling the expression of senescence-associated genes upon infestation.

### Stress signalling in aphid-attacked plants is moderately weaker in the JA-deficient mutant

Proteins involved in the perception of stress and transduction of signals play an important role in the initiation of defence responses [7]. After 72 h of sustained aphid infestation a large number of genes coding for proteins involved in calcium signalling, signal transduction and redox changes were up-regulated in the aphid-attacked wt plants.

Similar responses were also triggered in the *aos *mutant but the average intensity of gene regulation was slightly lower compared to wt. Only transcripts associated with redox processes responded to infestation with higher average induction in *aos *than in wt plants. These observations indicate that the JA-deficient mutant is not impaired in the perception and transduction of signals during infestation and that JA signalling plays only a partial role in the activation of these processes.

In contrast, the aphid-triggered responsiveness of genes connected to stress signalling was reduced in the *fou2 *mutant. The GO category denoted "regulation of biological processes", which included "regulation of response to stimuli" and "signal transduction", was statistically significantly enriched as indicated by the GO Term Enrichment analysis of genes that were less responsive to infestation in the *fou2 *mutant (Figure 4A). Signal transduction, calcium signalling and redox gene categories were also abundantly represented among genes that were less induced by infestation in *fou2 *than in wt (Figure 3). The expression of 45, 20 and 16 genes related to respective functional categories were either not changed, changed to a lesser extent than in wt or were oppositely regulated in response to infestation in *fou2 *plants (Additional file 7 Table S5). However, some of these genes were up-regulated in the non-challenged *fou2 *mutant in comparison to wt [e.g. *calcium-binding EF-hand*: *CML38, CML41, CaMBP25*, *blue copper-binding*, *DSBA oxidoreductase family gene, monodehydroascorbate reductase *(*MDAR1*)*, glutaredoxin family protein *(*GRX480*)*, thioredoxin H-type 5 *(*TRX5*)*, FAD-linked oxidoreductase, peroxidase 32 precursor *(*PER32*)]. Thus, processes connected to the perception and transduction of signals seem to be imbalanced in the non-challenged *fou2 *mutant and their activation upon aphid infestation might be impaired.

### Changed JA status leads to the induction of genes connected to transport and cell wall modifications

Both *aos *and *fou2 *mutants responded to infestation by up-regulation of genes linked to transport, while the average expression profile of these genes in wt plants remained unchanged after *B. brassicae *attack. GO Term Enrichment analysis indicated that mainly GO terms connected to boron and lipid transport were effected in *fou2 *(Figure 4B). It is possible that in response to infestation, plants in which the JA synthesis rate is somehow disturbed (either from a lack of JA in *aos *or an overproduction of JA-related compounds in *fou2*) try to compensate for unbalanced JA signalling by induction of cellular transport.

Interestingly, some genes whose products are involved in cell wall modification were differentially regulated upon infestation in the mutant plants in comparison to wt. These genes also make a considerable contribution to the set of all genes that were more induced by aphid attack in *aos *and *fou2 *mutants than in wt (Figure 3). As revealed by AmiGO Term Enrichment analysis, GO terms connected to cell wall organization and aminoglycan and polysaccharide metabolic processes are overrepresented in the set of genes that were more induced by aphid attack in the *fou2 *mutant (Figure 4B). Generally these genes were slightly down-regulated in the aphid-challenged wt plants, not responsive in infested *aos *and slightly up-regulated in infested *fou2*. Their expression was not changed in aphid-free mutants as compared to wt. Thus, it seems that hyper-activation of the JA signalling pathway in the *fou2 *mutant might cause some changes in cell walls that do not occur in the infested wt plants.

### The *fou2 *mutation increases plant resistance to *Brevicoryne brassicae *by a mechanism other than feeding deterrence

**Figure 5:**
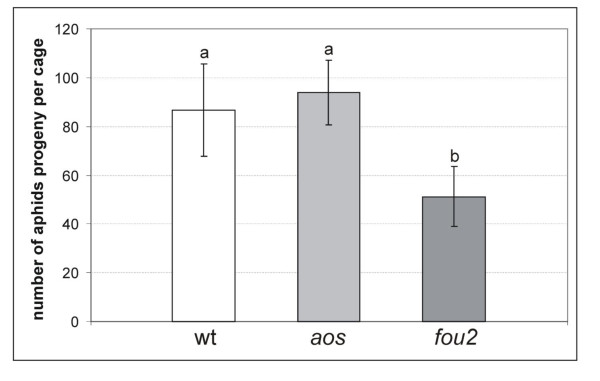
**Asexual fecundity of *Brevicoryne brassicae *on wt Col-0, *aos *and *fou2 *plants**. Initially two first instar nymphs were placed on each of the three plants kept in the same cage during the experiment. In total, 11 cages (33 plants) of each of the genotypes were used in the experiment. Bars represent an average number (+/- SD) of aphid progeny per cage after 13 days. Different letters indicate statistically significant differences between the numbers of newborn aphids on different genotypes as revealed by a two-tailed Wilcoxon rank sum test.

**Figure 6:**
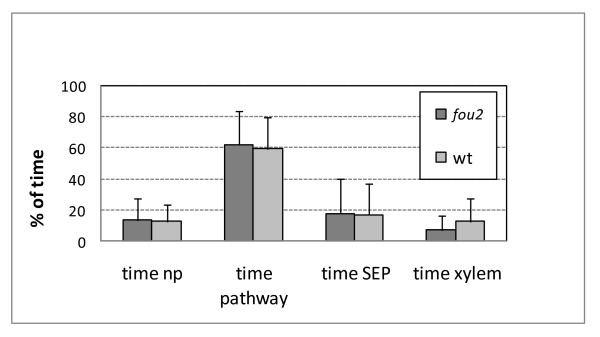
**The relative amount of time spent on different feeding behaviours by *Brevicoryne brassicae *on *fou2 *and wt plants as revealed by Electrical Penetration Graph recordings during 8 hours-long experiments**. Abbreviations: time np, time spent on plant without physical contact between stylet and plant tissue; time pathway, time spent on penetration of plant tissue by stylet; time SEP, time spent on feeding from sieve element; time xylem, time spent on feeding from xylem. None of the observed differences was statistically significant (Wilcoxon rank sum test).

The relative susceptibility of *aos*, *fou2 *and wt plants to infestation with *B. brassicae *was evaluated in aphid fitness experiments. First instar nymphs were placed on each of the three genotypes and their asexual fecundity was monitored simultaneously. After 13 days the number of offspring did not differ significantly between *aos *and Col-0 plants. However, aphid fecundity on the *fou2 *mutant was significantly lower when compared to the fecundity observed on *aos *and wt plants (Figure 5). To further investigate whether some anti-xenotic (feeding deterrent) factors are involved in the observed resistance of *fou2 *to *B. brassicae*, we employed the Electrical Penetration Graph (EPG) technique. EPG allowed us to monitor and compare the amount of time the aphids spent on various activities connected to the penetration of plant tissue and ingestion of phloem sap on *fou2 *mutants and wt plants. The electrical waveforms, corresponding to non-probing (when the stylet does not have any contact with plant tissue), pathway (where the stylet is manoeuvred through plant tissue accompanied by sheath salivary discharges), the sieve element phase (called SEP, when the stylet is located in a sieve element), and xylem phase (when the stylet is located in a xylem cell) were recorded for 8 h and categorized according to known wave patterns corresponding to each activity. The average time spent on each activity was calculated separately for aphids feeding on *fou2 *and wt plants. The time aphids spent on non-probing, pathway, and SEP was similar in the case of *fou2 *and wt plants (Figure 6). As phloem sap uptake from *fou2 *mutants was not restricted, we conclude that feeding deterrence was not the factor limiting *B. brassica*e population size on *fou2 *plants.

## Discussion

### JA signalling contributes to aphid-triggered regulation of a wide range of genes

Several experiments have proven that infestation with phloem feeders leads to extensive transcriptional reprogramming of the attacked plants. Gene expression changes manifested in the current experiment in wt plants 72 h after infestation with *B. brassicae *correspond well to the changes previously observed in different *A. thaliana *ecotypes attacked by green peach aphid (*Myzus persicae*) or *B. brassicae *[9]. Although such a long period of infestation may cause secondary effects linked to withdrawal of significant amounts of amino acids and sugars contained in the phloem sap, most of the transcriptional changes were similar to those observed in earlier phases of infestation (e.g. 48 h, 24 h, or even 12 h) [7]. This indicates that there is no dramatic change in the type of responses activated 72 h after aphid attack as compared to earlier stages of infestation.

Jasmonates are physiological signals for defence. The enhanced production of JA in response to pathogen and insect attack regulates expression of many defence-related genes and may induce broad-spectrum resistance [32]. Interestingly, many of the genes that were up-regulated in response to infestation in wt plants have shown similar induction in the non-challenged *fou2 *mutant. Characterization of *fou2 *by Bonaventure and co-workers revealed strong induction of defensive mechanisms resulting from overproduction of JA [33]. Other studies have demonstrated that the application of methyl jasmonate also causes activation of the JA pathway and similar up-regulation of genes connected to defence, responses to oxidative stress, and cell wall modification [34-36]. Similar changes have also been detected at the protein level [37]. Although plants that are deficient in the production of JA do not show any symptoms of disease when grown under laboratory conditions, our study clearly shows that lack of JA negatively influences the basal expression of a wide range of genes. As expected, many of these genes encode proteins that are directly or indirectly involved in plant defence. A number of JA-dependent defence-related transcripts were induced in wt plants during *B. brassicae *attack, but only a few of these were activated in the challenged *aos *mutant, which showed that the regulation of these genes upon aphid attack is primarily controlled through JA signalling. Aphid-mediated induction of many other genes was clearly affected by the *aos *mutation as well. Although the transcription of many of these genes was apparently not dependent on the JA status in non-challenged plants, JA-derived signals comprised a significant contribution to their regulation in infested plants. Aphid-induced changes in the expression of a number of transcription factors such as *WRKY*, *C2H2 zinc fingers*, BTB and TAZ domain containing proteins and *ERFs *were weaker in *aos *than in wt, indicating the importance of JA for their induction. WRKY transcription factors are important in SA-dependent defence and some are implicated in cross-talk between JA and SA signalling [38]. Transcription factors containing ethylene responsive domains have been shown to be regulated by JA [39,40] and to participate in plant stress responses [41-43]. They may integrate ET- and JA-derived signals, possibly by interaction with the GCC box in the promoter region of JA-regulated genes [44] and act as both positive and negative regulators of transcriptional changes [39]. Transcription factors such as AP2-domain protein ERF018/ORA47, ZAT10 and AZF2 have been previously identified as both positive and negative regulators of JA signalling [45]. However, their involvement in the activation of plant defence has not been assessed yet. Strong up-regulation of these genes in wt plants attacked by *B. brassicae *suggests that they play an important role in defence against aphids. The regulatory function of BTB and TAZ domain containing proteins has not been established yet, but BTB and TAZ domain protein (BT2) have been identified as essential components of the TELOMERASE ACTIVATOR1 (TAC1)-mediated telomerase activation pathway [46]. Telomerase activity is high in plants in rapidly dividing cells and reproductive organs. The induction of BT2 and BT5 in the non-challenged *aos *plants suggests that these genes are under negative regulation of JA. All five BTB and TAZ proteins (BT1-BT5) are known to be readily induced by H_2_O_2 _and SA treatments [47].

The glutaredoxin family protein GRX480, whose induction was eliminated in the infested *aos *plants, was recently identified as a regulator of JA/SA cross talk. It interacts with TGA transcription factors to antagonize expression of JA-responsive genes in an NPR1-dependent manner [48]. Our results indicate that the induction of GRX480 upon *B. brassicae *attack is dependent on JA levels.

The expression of *EDS5 *in both non-challenged and aphid-attacked plants shows that JA levels also influence it. This is in contrast to previous reports, which describe solitary SA signalling based regulation of the *EDS5 *gene [49]. Our results suggest that regulation of *EDS5 *is more complex than previously thought.

### Additional signals are involved in regulation of the response to *B. brassicae *infestation

Some genes, whose expression in non-challenged plants was clearly dependent on JA responded to infestation in the *aos *mutant despite the lack of JA-derived signals, even though their induction was not as extensive as the induction observed in wt plants. This indicates that, in addition to JA, some other signalling mechanisms are involved in the regulation of these transcripts upon *B. brassicae *infestation. It is well established that the activation of invader-specific responses in plants attacked by insects is mediated by cross-talk between different signalling pathways [38]. In the case of insect infestation, in addition to JA, phytohormones such as salicylic acid (SA), ethylene (ET) and abscisic acid (ABA) play major roles in coordinating the induction of appropriate defences [26,50]. Thus SA, ET or ABA are likely regulators of the defence responses in the absence of JA for genes such as *trypsin inhibitors *(*ATTI1 *and *At1g73260*), *TAT3*, *CYP79B2, PR4 *or *ASA1*.

### Induction of JAZ repressors desensitizes *fou2 *response to *B. brassicae *attack

The transcriptional profile of the non-challenged *fou2 *genotype mimics the profile of wt plants that manifest induced defence [33]. In our studies many of the genes that have been shown to be involved in the response to aphid attack in wt plants were up-regulated in the non-challenged *fou2 *mutant, often showing similar or stronger intensity of changes compared to attacked wt plants (Table 1 and Additional file 7 Table S5). A similar induction of transcription factors and defence-related genes was observed by Bonaventure and co-workers [33]. However, in contrast to the previously observed reaction of *fou2 *to wounding [17], further induction of these transcripts upon infestation was much weaker than observed in wt plants. A similar lack of stress responses resulting from prolonged high endogenous JA levels was observed in potato plants subjected to wounding and water stress. Although several of the genes involved in JA biosynthesis are induced by JA thereby creating a positive feedback loop [51], there exists also a negative regulatory feedback loop protecting the plants from the adverse effects of their own defence. The constitutive up-regulation of the JA synthesis pathway in the *fou2 *mutant probably triggers this negative feedback loop, leading to desensitization of processes involved in the activation of the aphid-induced defence. JAZ family proteins act to repress transcription of JA-inducible genes and thus modulate JA-mediated plant responses [52,53]. The high induction of several *JAZ *genes in the *fou2 *mutant (Additional file 3 Table S1) indicates activation of the desensitization mechanism and may explain the reduced responsiveness of *fou2 *plants challenged with *B. brassicae*. The negative regulation of JA responses is delayed and takes effect some time after the proceeding induction [45]. The hyper activation of JA biosynthesis genes in *fou2 *plants shortly after mechanical wounding that was observed by Bonaventure and co-workers [17] was not observed by us after 72 h of sustained *B. brassicae *infestation. This might be due to a stealthy manner of aphid feeding that causes only minimal tissue damage. The induction of the wound-specific JA responses in aphid-infested plants is therefore much weaker than in mechanically wounded plants. In addition, the high level of JAZ repressors may also tune the JA-regulated transcriptional changes in the aphid-attacked *fou2 *plants after 72 h.

### Aphid fitness is comparable on wt and *aos *genotypes but reduced on *fou2*

Despite the reduced responsiveness of a wide range of defence-linked genes in the *aos *mutant, we did not observe any improvement in aphid fitness in comparison to wt plants. This may seem surprising as JA signalling seems to be important for plant defence mechanisms induced upon infestation. In contrast to our results, Ellis and co-workers observed increased growth of green peach aphid (*Myzus persicae*) populations on the *coi1-16 *mutant that had defects in JA signalling [13]. However the *coi1-16 *line carries an additional mutation that might have influenced *M. persicae *responses observed by Ellis and co-workers. This mutation lies in the *PENETRATION2 *(*PEN2*) gene encoding a glycoside hydrolase and renders the PEN2 protein with highly reduced stability [54]. PEN2 is required for indole glucosinolate-dependent pathogen-induced callose deposition [55]. As accumulation of callose is one of the defence mechanisms against aphid infestation [7], the *pen2-4 *mutation, present in *coi1-16 *line, may contribute to the increased susceptibility of *coi1-16 *plants to infestation with *M. persicae*.

It is also conceivable that the expressional changes of JA-regulated genes observed by us in the aphid-infested *aos *mutant were sufficient to sustain the same level of aphid resistance/susceptibility as is present in wt plants. It should be noted that many genes known to be regulated by SA, ABA or auxin signalling were up-regulated in *aos *plants. Several of these can be involved in defence against *B. brassicae *infestation and influence aphid fitness.

As revealed by the insect fitness tests, physiological changes resulting from the *fou2 *mutation render plants more resistant to infestation than wt, despite the reduced intensity of the aphid-induced responses. As the observed resistance was not based on feeding deterrence, it is most probably based on antibiosis. Various defence-related responses that are constitutively activated in *fou2 *plants, e.g. high expression of plant defensin proteins (PDFs), pathogenesis-related proteins (PR) or protease inhibitors, can exhibit an antibiotic effect on insect pests. The latter, for example, can disturb digestion and absorption of food in the insect gut [27]. Moreover, the high activity of LOX enzyme in *fou2 *plants can increase production of reactive lipid peroxides, cause oxidative damage to the insect gut and significantly decrease the nutritive quality of dietary proteins [56]. It should be noted, however, that the mechanism responsible for the manifestation of the *fou2 *phenotype is not fully understood. Therefore, we cannot eliminate the possibility that other, unknown, features of *fou2 *could play a role in mediating aphid resistance.

## Conclusions

A comparison of transcriptional profiles of non-challenged *aos*, *fou2 *and wt plants allowed us to identify more than 200 genes whose expression profiles in non-challenged plants were dependent on endogenous jasmonate status. Most of these transcripts were up-regulated in *fou2 *and down-regulated in *aos *mutants, which points to a positive regulatory function of JA-derived compounds. Many of the jasmonate-dependent genes were connected to regulation of transcription, defence responses, redox balance and cell wall modification.

Upon infestation with *Brevicoryne brassicae*, the responsiveness of many genes was changed in *aos *and *fou2 *plants. Genes attributed to GO categories connected to the regulation of transcription and responses to stress were generally less induced in both mutants. In contrast, transcripts classified as involved in cell division and development, cell wall modification and transport were more induced or not as much down-regulated in the mutants compared to wt. The observed changes in aphid-mediated responsiveness of *aos *had, however, no noticeable impact on aphid fitness. This may indicate that the induced responses, although weaker than in wt, were strong enough to keep the same level of resistance. Alternatively, responses were mainly induced locally, so that the aphids could benefit from frequent changes of feeding places. In the *fou2 *mutant, several genes involved in defence against *B. brassicae *were induced in non-challenged plants. As a consequence, the transcriptional profile of non-challenged *fou2 *resembled the aphid-induced profile of wt. Although additional *B. brassicae *mediated regulation of already induced genes was limited, the aphids' reproduction rate was negatively influenced by the *fou2 *mutation. As an array of defensive responses is constitutively activated in *fou2 *plants, the feeding aphids could not move to a leaf area where the response was not induced, as they could in the case of wt plants.

Our results indicate that JA-regulated responses are important in defining susceptibility of a plant to infestation with aphids. As shown in this study, JA-derived compounds are powerful regulators of a range of defensive responses exhibited by plants attacked by aphids.

## Methods

### Plant material

The *Arabidopsis thaliana *Columbia-0 ecotype (Col-0) single seeds line used in the experiment has been derived from seeds produced by Lehle Seeds (Round Rock, USA; Catalogue No. WT-2-8, Seed Lot No. GH195-1). The *aos *mutant was the one described in [15]. The *fou2 *mutant was kindly donated by Prof. Edward Farmer (University of Lausanne, Switzerland). Both mutants are in Col-0 background. Seeds were sterilized according to standard procedures and plants were initially grown aseptically on agar medium containing MS basal salt mixture (Sigma), 3% (v/w) sucrose, and 0.7% (v/w) agar (pH 5.7) to assure uniform germination. After 15 days, seedlings were moved to 6 cm diameter pots (3 seedlings per pot) filled with a sterile soil mix (1.0 part soil, and 0.5 part horticultural perlite). Plants were kept in growth chambers Vötsch VB 1514 (Vötsch Industrietechnik GmbH, Germany) under the following conditions: a 8/16 h (light/dark) photoperiod at 22°C/18°C, 40%/70% relative humidity, and 70/0 μmol m^-2^s^-1 ^light intensity. A short time day was applied to prevent plants from bolting. For aphid fitness experiments, plants were sown directly to pots with soil (one plant per pot) and kept in chambers under a 16/8 h (light/dark) photoperiod.

### Insects

*Brevicoryne brassicae *was reared on *Brassica napus *or *Brassica oleracea *plants in a growth chamber with a 16/8 h (light/dark) photoperiod at 22°C/18°C, 40%/70% relative humidity, and 70/0 μmol m^-2^s^-1 ^light intensity.

### Infestation experiments

Thirty-two-day-old plants (17 days after transferring to soil) had 8 fully developed leaves. Each plant was infested with 32 wingless aphids (4 per leaf), which were transferred to leaves with a fine paintbrush. Infested plants and aphid-free controls were kept in plexiglass cylinders as described in [9]. Plants were harvested 72 h after infestation between the 6th and 8th hour of the light photoperiod. Four biological replicates were run, each sampled from 15 individual plants. Whole rosettes were cut at the hypocotyls and aphids were removed by washing with Milli-Q-filtered water. Harvested material was immediately frozen in liquid nitrogen.

### RNA isolation, cDNA synthesis and microarray experiments

All procedures were done as described in [7]. Custom-designed, full genome *Arabidopsis *oligonucleotide microarrays printed at the Norwegian Microarray Consortium (Trondheim, Norway) were used in all experiments.

### Quantitative real-time PCR

For qRT-PCR analysis, the total RNA was DNAse treated using DNA-free™ Kit (Applied Biosystems), while the QuantiTect^® ^kit (QIAGEN) was used for cDNA synthesis. A LightCycler 480 System and the corresponding SYBR Green I Master mix (Roche Diagnostics GmbH) were used in a three-step programme including (1) preincubation at 95°C for 5 min; (2) 40 cycles of amplification consisting of 95°C for 10 s, 55°C or 60°C for 10 s and 72°C for 10 s; and (3) melting curve analysis by heating from 65°C to 97°C with a ramp rate of 2.2°C/s. Each 20 μl reaction contained 0.5 μM of each forward and reverse primer (for gene-specific primer sequences used in qRT-PCR, see Additional file 9 Table S7), and cDNA quantity corresponding to 50 ng of RNA. LinRegPCR software [57] was used to determine the PCR reaction efficiency for each sample and the efficiencies for each primer set were calculated by averaging the efficiency values obtained from the individual samples. Relative expression ratios of the targeted genes were calculated and normalized to *TIP41*-like gene (At4g34270) [58] with the use of REST 2008 software [59]. The qRT-PCR analysis was performed with the use of three biological replicates.

### Statistical analysis of microarray data

The microarray experiment was a 2-by-3 factorial, with the factors as plant type (wt, *aos *mutant or *fou2 *mutant) and treatment (infested or not infested). Each experimental condition, i.e. each combination of factors, was represented by four biological replicates. Seven different direct comparisons of the experimental conditions, using four replicates (each representing 15 individual plants) for each comparison, were made with the use of microarray data sets. However, only data from microarrays with very good technical quality were used for further analyses. (Figure 1 shows the direct comparisons that were made and the comparisons for which only three replicates were of good enough technical quality). Note that using this setup means that the same biological replicate will occur on two different microarrays. Also note that experimental conditions that were not compared directly can still be contrasted, but with lower efficiency than the direct comparisons.

The microarray data for each array were filtered and normalized as discussed in [7]. To make statistical inferences about differential regulation between experimental conditions, the limma package [60] was used. In each comparison of experimental conditions a q-value [61] was calculated for each gene. For a gene to be considered differentially regulated at a statistically significant level, its q-value had to be lower than 0.05. In effect this controlled the false discovery rate (FDR) [62-64] of the comparison at a 0.05 level.

### Aphid fitness experiments

*B. brassicae *fitness on *aos *and *fou2 *mutants in comparison to wt Col-0 was evaluated in experiments assessing aphid asexual fecundity. Two first instar nymphs were placed on each plant and plants were placed in plexiglass cages (3 plants per cage). Eleven cages (33 plants) were used for each genotype tested. After 13 days, aphid progeny numbers in each cage were counted. To compare aphid counts for the different plant types, a two-tailed Wilcoxon rank sum test was used with a significance level of 0.05.

### Electrical Penetration Graph

The EPG technique was used to monitor aphid feeding behaviour [65]. An eight-channel GIGA-8 direct current amplifier (Wageningen University, The Netherlands) was used for simultaneous recordings of eight individual wingless *Brevicoryne brassicae *aphids feeding on eight plants (4 wt plants and 4 *fou2 *mutants). The aphids originated from a colony kindly donated by Prof. Gary Thompson (Oklahoma State University) propagated on *Brassica oleracea *plants. Before the start of an experiment, the aphids were starved for 4 h and immediately before wiring, an individual aphid's dorsum was cleaned of wax with the help of a paintbrush hair, and a thin gold wire (12.7 μm diameter, 2-4 cm long) was glued to the dorsum with silver paint (Ted Pella). The other end of the wire was connected to an EPG probe and an output wire from the EPG monitor was inserted into the soil in which the plant was rooted. Plants used in EPG experiments were 3 to 4 weeks old, and did not reach the bolting stage. During experiments plants and insets were kept inside a Faraday cage at constant light conditions and 22°C. The waveform recordings were analysed using the EPG analysis software PROBE 3.0 (W.F. Tjallingii, Wageningen University, The Netherlands). The experiments were repeated several times to obtain a total of 30 biological replicates for *fou2 *and 34 for wt. A Wilcoxon rank sum test was used to compare the amount of time *B. brassiae *spent on different feeding behaviours as measured with EPG.

## Authors' contributions

AK, PW, TSJ and AMB designed the study. AK, DHTT and JT performed infestation experiments for microarray analyses. TSJ performed statistical analysis of transcriptional data. AK, DHTT and PW analysed the transcriptional data. AK performed aphid fitness and EPG experiments and analysed EPG data. JCR contributed with EPG equipment and coordinated EPG experiments. AK, DHTT and PW wrote the manuscript. AMB coordinated the study and completed the submission. All authors read and approved the final version of manuscript.

## Supplementary Material

Additional file 1 **Figure S1. Consequences of the *aos *and *fou2 *mutations on jasmonic acid biosynthesis *in planta***. Click here for file

Additional file 2 **Figure S2. Verification of microarray data by quantitative RT-PCR**. Click here for file

Additional file 3 **Table S1. List of genes whose expression in non-challenged plants was positively influenced by jasmonates**. Gene expression values for which regulation was not statistically significant are shaded in grey. Click here for file

Additional file 4 **Table S2. List of genes whose expression in non-challenged plants was negatively influenced by jasmonates**. Gene expression values for which regulation was not statistically significant are shaded in grey. Click here for file

Additional file 5 **Table S3. List of genes that were less induced in response to *B. brassicae *infestation in *aos *than in wt plants**. Gene expression values for which regulation was not statistically significant are shaded in grey. Click here for file

Additional file 6 **Table S4. List of genes that were more induced in response to *B. brassicae *infestation in *aos *than in wt plants**. Gene expression values for which regulation was not statistically significant are shaded in grey. Click here for file

Additional file 7 **Table S5. List of genes that were less induced in response to *B. brassicae *infestation in *fou2 *than in wt plants**. Gene expression values for which regulation was not statistically significant are shaded in grey. Click here for file

Additional file 8 **Table S6. List of genes that were more induced in response to *B. brassicae *infestation in *fou2 *than in wt plants**. Gene expression values for which regulation was not statistically significant are shaded in grey. Click here for file

Additional file 9 **Table S7. Primers used in quantitative RT-PCR analysis**. Click here for file
