# Supplementary material for: Testing the importance of jasmonate signalling in induction of plant defences upon cabbage aphid (Brevicoryne brassicae) attack
Source: BMC Genomics. 2011 Aug 19;12:423. doi: 10.1186/1471-2164-12-423 (PMC3175479; doi:10.1186/1471-2164-12-423)
Supplement: Additional file 1 — Figure S1. Consequences of the aos and fou2 mutations on jasmonic acid biosynthesis in planta. [file 1471-2164-12-423-S1.DOC]

**Additional file Figure S1.** Consequences of the *aos* and *fou2* mutations on jasmonic acid biosynthesis *in planta*. The lack of AOS enzyme in the *aos* mutant results in plants compromised in the synthesis of jasmonates. In the *fou2* mutant increased activity of LOX and AOS enzymes leads to the accumulation of higher amounts of OPDA and JA. The biosynthetic pathway scheme is simplified for clarity of presentation. Abbreviations: 13-LOX, 13-lipoxygenase; 13-HPOT, 13-hydroperoxylinolenic acid; AOS, allene oxide synthase; AOC, allene oxide cyclase; OPDA, 12-oxo-phytodienoic acid; JA, jasmonic acid.
