# Supplementary material for: Testing the importance of jasmonate signalling in induction of plant defences upon cabbage aphid (Brevicoryne brassicae) attack
Source: BMC Genomics. 2011 Aug 19;12:423. doi: 10.1186/1471-2164-12-423 (PMC3175479; doi:10.1186/1471-2164-12-423)
Supplement: Additional file 2 — Figure S2. Verification of microarray data by quantitative RT-PCR. [file 1471-2164-12-423-S2.DOCX]

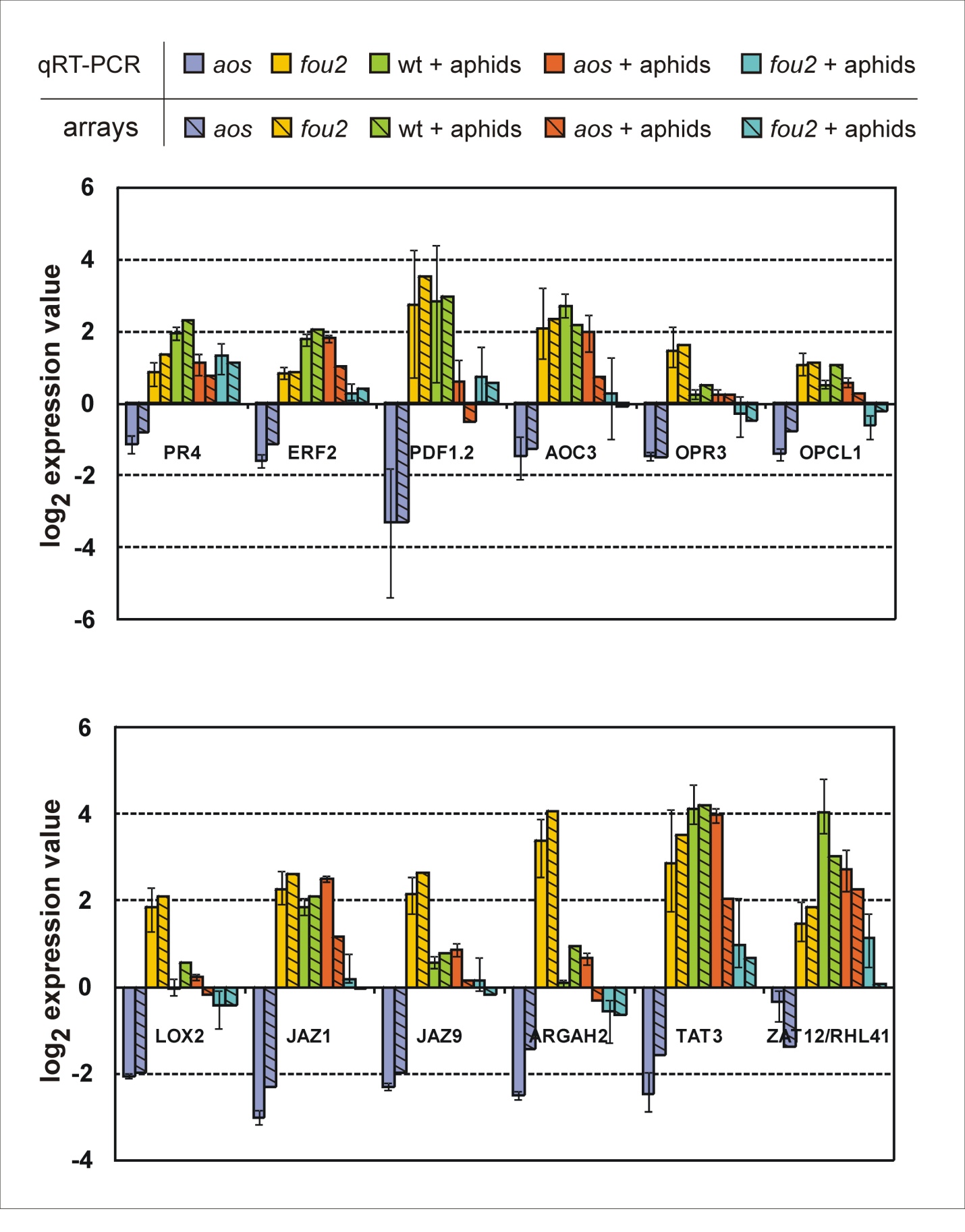


**Additional file Figure S2.** Verification of microarray data by quantitative RT-PCR. Bars represent averages of log_2_ transformed expression values +/- standard errors for three biological replicates. Abbreviations: *aos*, changes in gene expression profile in the *aos* mutant in comparison to wt as revealed by qRT-PCR and microarray results; *fou2*, changes in gene expression profile in the *fou2* mutant in comparison to wt as revealed by qRT-PCR and microarray results; wt + aphids, changes in gene expression mediated by *B. brassicae* attack in wt plants as revealed by qRT-PCR and microarray results; *aos* + aphids, changes in gene expression mediated by *B. brassicae* attack in the *aos* mutant as revealed by qRT-PCR and microarray results; *fou2* + aphids, changes in gene expression mediated by *B. brassicae* attack in the *fou2* mutant as revealed by qRT-PCR and microarray results; PR4, *PATHOGENESIS-RELATED 4*; ERF2, *ETHYLENE RESPONSIVE TRANSCRIPTION FACTOR 2*; PDF1.2, *PLANT DEFENSIN 1.2*; AOC3, *ALLENE OXIDE CYCLASE 3*; OPR3, *OPDA REDUCTASE 3*; OPCL1, *OPC-8:0 CoA LIGASE 1*; LOX2, *LIPOXYGENASE 2*; JAZ1, *JASMONATE-ZIM-DOMAIN PROTEIN* *1*; JAZ9, *JASMONATE-ZIM-DOMAIN PROTEIN 9*; ARGHA2, *ARGININE AMIDOHYDROLASE 2*; TAT3, *TYROSINE AMINOTRANSFERASE 3*; ZAT12/RHL41, *RESPONSIVE TO HIGH LIGHT 41*.
