# Supplementary material for: Testing the importance of jasmonate signalling in induction of plant defences upon cabbage aphid (Brevicoryne brassicae) attack
Source: BMC Genomics. 2011 Aug 19;12:423. doi: 10.1186/1471-2164-12-423 (PMC3175479; doi:10.1186/1471-2164-12-423)
Supplement: Additional file 9 — Table S7. Primers used in quantitative RT-PCR analysis. [file 1471-2164-12-423-S9.DOC]

Additional file Table S7. Primers used in quantitative RT-PCR analysis.

| **gene** | **forward primer** | **reverse primer** |
| --- | --- | --- |
| ***PR4*** | GAAGATCAGACTTAGCATAACC | TTGTTGGTCGGAGAACAGTAGT |
| ***ERF2*** | ACTACGCTTTGTTGGAGTCGAT | GTACACCAACATGTCCTCTGAA |
| ***PDF1.2*** | GCAAGAATCAGTGCATTAACCT | TACACACGATTTAGCACCAAAG |
| ***AOC3*** | TTCTTAAACTCGGCAAGAAACC | CTCTTTTCAGGAACGTGTTGGA |
| ***OPR3*** | CATGCAGTGTATCAACCTAATG | CCAGCTCGAATCGCATTCAAAG |
| ***OPCL1*** | GTGAATCGATTTGGATCTGATG | GTAGATCCGTAAGCGAGTAATC |
| ***LOX2*** | TGGTAAACTATGGAGGTTTGAC | GCAAATGGATAGTCTGGTATCG |
| ***JAZ1*** | CACAGACGTGTAGTCGATTGAG | GACGTGAGTTGCCTAAAGTTCC |
| ***JAZ9*** | TTCAATGCAGCTCCTCGTAACA | TTTGCGCTTCTCCAAGAACCGA |
| ***ARGAH2*** | GCAGAACCGTGTCATTGATGCT | ACTCCAAGAAGAGCTGTTGTAG |
| ***TAT3*** | TTGATTTCTGCACGAAGCTAGT | TTGTACCACTGATTCGTCGGTT |
| ***ZAT12*** | TGAATCTCAAGTTGGAGCTTGG | TTCCTAAATCTGCGGATGTATG |
| ***TIP41-like*** | GTGAAAACTGTTGGAGAGAAGCAA | TCAACTGGATACCCTTTCGCA |
